# Comparative Analysis of Different Inbred Chicken Lines Highlights How a Hereditary Inflammatory State Affects Susceptibility to Avian Influenza Virus

**DOI:** 10.3390/v15030591

**Published:** 2023-02-21

**Authors:** Karen J. Bryson, Samantha Sives, Hui-Min Lee, Dominika Borowska, Jacqueline Smith, Paul Digard, Lonneke Vervelde

**Affiliations:** 1 Division of Immunology, The Roslin Institute & R(D)SVS, University of Edinburgh, Easter Bush, Edinburgh EH25 9RG, UK; 2 Division of Virology, The Roslin Institute & R(D)SVS, University of Edinburgh, Easter Bush, Edinburgh EH25 9RG, UK; 3 Division of Genome Biology, The Roslin Institute & R(D)SVS, University of Edinburgh, Easter Bush, Edinburgh EH25 9RG, UK

**Keywords:** chicken, avian influenza virus, immune response, inflammation, genetic variation

## Abstract

Evidence suggests that susceptibility to avian influenza A virus in chickens is influenced by host genetics, but the mechanisms are poorly understood. A previous study demonstrated that inbred line 0 chickens are more resistant to low-pathogenicity avian influenza (LPAI) infection than line CB.12 birds based on viral shedding, but the resistance was not associated with higher AIV-specific IFNγ responses or antibody titres. In this study, we investigated the proportions and cytotoxic capacity of T-cell subpopulations in the spleen and the early immune responses in the respiratory tract, analysing the innate immune transcriptome of lung-derived macrophages following in vitro stimulation with LPAI H7N1 or the TLR7 agonist R848. The more susceptible C.B12 line had a higher proportion of CD8αβ^+^ γδ and CD4^+^CD8αα^+^ αVβ_1_ T cells, and a significantly higher proportion of the CD8αβ^+^ γδ and CD8αβ^+^ αVβ_1_ T cells expressed CD107a, a surrogate marker of degranulation. Lung macrophages isolated from line C.B12 birds expressed higher levels of the negative regulator genes *TRIM29* and *IL17REL,* whereas macrophages from line 0 birds expressed higher levels of antiviral genes including *IRF10* and *IRG1*. After stimulation with R848, the macrophages from line 0 birds mounted a higher response compared to line C.B12 cells. Together, the higher proportion of unconventional T cells, the higher level of cytotoxic cell degranulation ex vivo and post-stimulation and the lower levels of antiviral gene expression suggest a potential role of immunopathology in mediating susceptibility in C.B12 birds.

## 1. Introduction

Waterfowl are the natural reservoir host of Avian Influenza A virus (AIV) [1], with many species of birds deemed susceptible. The majority of circulating AIV strains are classified as low-pathogenicity avian influenza (LPAI) pathotypes, but viruses of the H5 and H7 haemagglutinin subtypes can evolve into highly pathogenic (HPAI) strains that cause much higher mortality in gallinaceous poultry [1]. The introduction of even the LPAI form of the virus to commercial poultry can have a severe economic impact, through a reduction in egg production and sometimes the mortality of birds, along with the detection of the virus often resulting in flock culling to prevent further transmission [2,3]. Alongside welfare and economic consequences, the zoonotic potential of AIV and its potential role in a future pandemic escalate the virus to global importance [4]. The urgency of effective control measures, together with an evaluation of the animal host-specific factors related to infection and zoonotic potential, has long been acknowledged. Consequently, it is a high research priority of the World Health Organisation in order to reduce the risks of an emerging pandemic influenza originating from avian viruses [5].

The resistance and susceptibility to AIV strains in chickens have been associated with differences in the genetic diversity of the birds. While major histocompatibility (MHC) class I (haplotype B21) was linked to survival following an outbreak in Thailand [6], non-MHC-related genes, such as Mx [7] and the IFITM gene family [8], may also influence resistance to AIV [9]). Other genes exerting an influence on physiology, rather than directly on immune responses and their associated downstream signalling pathways, have also been identified as contributors to resistance, such as those impacting oxygen transport [10]. After the outbreaks of H5N2 HPAI virus in commercial layer flocks in the US, Drobik-Czwarno et al. (2018) [11] analysed the genetic basis of the resistance of survivors and confirmed that resistance to HPAI is a complex, polygenic trait and that mechanisms of resistance may be population-specific.

Genetically (partially) inbred lines differing in susceptibility to AIV can reveal genomic regions, candidate genes and SNPs associated with survival and resilience [10,11]. A study by Ruiz et al. (2016) [12] using the LPAI virus infection of chicken MHC I inbred line C.B12 (B12 haplotype) and line 0 (B21 haplotype) demonstrated that line 0 birds were more resistant to infection, in that animals shed the virus from the oropharynx but not from the cloaca following intranasal infection. Based on the number of AIV-specific IFNγ-secreting cells and AIV-specific IgM- and IgY-secreting B cells in the spleen at 1 and 2 weeks post-infection, the authors concluded that the underlying mechanisms mediating a greater resistance to AIV may not solely be based on differences in adaptive immune responses. Hereditary differences between the bird lines influencing the kinetics of the early immune response may also have contributed to their differential responses to LPAI [12]. Here, we used the same chicken lines, C.B12 and line 0, to investigate the proportions and cytotoxic capacity of T-cell subpopulations in the spleen and the early immune responses in the respiratory tract by an analysis of the innate immune transcriptome of macrophages isolated from the lung, after in vitro stimulation with LPAI H7N1 or a TLR7 agonist (R848). The more susceptible birds had a higher proportion of unconventional T cells and a higher level of cytotoxic cell degranulation ex vivo and post stimulation, which suggest a potential role of immunopathology in mediating susceptibility. In addition, the innate antiviral gene expression was lower, whereas the expression of negative regulator genes was higher, in susceptible C.B12 birds.

## 2. Materials and Methods

### 2.1. Chicken Lines

Line 0 (B21 haplotype) and line C.B12 (B12 haplotype) specific pathogen-free (SPF) layer-type birds were hatched and reared in floor pens at the National Avian Research Facility, The Roslin Institute, Edinburgh (UK). The chickens were housed in groups and received food and water ad libitum in premises licensed under a UK Home Office Establishment Licence (PEL 60/4604; UK Home Office Project Licence PE263A4FA) in full compliance with the requirements of the Animals (Scientific Procedures) Act 1986. The birds did not undergo any regulated procedures and were humanely culled in accordance with Schedule 1 of the Animals (Scientific Procedures) Act 1986 to provide material for the experiments described here.

### 2.2. Cell Preparation

Four-week-old line C.B12 and line 0 birds were culled by cervical dislocation, and their tissues were harvested. Single-cell suspensions were made by homogenising the spleen, passing the homogenate through a 70 μm cell strainer, and leukocytes were obtained by gradient purification with Lymphoprep 1.077 g/mL (Abbott Diagnostics, Maidenhead, UK) for 20 min at 400× *g* at room temperature. Cell numbers and viability were determined by Trypan Blue staining (Corning, St. David’s, UK), and cells were re-suspended in the appropriate media or buffer for the downstream application.

Lung macrophages were isolated from 8-week-old C.B12 and line 0 birds (n = five per line), as previously described [13]. The cells were cultured in cell-culture-treated 12-well plates (ThermoFisher Scientific (TFS), Paisley, UK) at 1.5 × 10^6^ cells/mL in a final volume of 2 mL with RPMI 1640 medium supplemented with 10% heat-inactivated FCS, 2 mM L-glutamine, 100 U/mL penicillin, 100 μg/mL streptomycin and 200 ng/mL CSF-1 at 41 °C and 5% CO_2_. After 48 h, the culture medium containing non-adhered cells was removed and replaced with fresh complete RPMI 1640 medium without CSF-1 for the infection (details below) [14].

### 2.3. Flow Cytometry

The cells were washed three times in FACS buffer (PBS supplemented with 0.5% bovine serum albumin and 0.01% sodium azide), incubated for 20 min at 4 °C with primary antibodies (Table 1), washed three times in FACS buffer, and incubated with the appropriate secondary antibodies for 20 min at 4 °C. The cells were washed three times in FACS buffer and resuspended in PBS, with SYTOX Blue (1:2000, TFS, UK) added prior to the acquisition as a marker of viability. Alternatively, following incubation with the appropriate primary and secondary antibodies and the washing steps, LIVE/DEAD fixable near infrared or violet dead stain kit reagents (1:3000, TFS) were added for 30 min at room temperature, and the cells were washed in PBS and fixed for 20 min in 1% paraformaldehyde at room temperature. The cells were then washed, resuspended in 300 μL FACS buffer and stored at 4 °C overnight prior to acquisition. Data were acquired using the LSRFortessa flow cytometer (BD Bioscience, UK) and analysed using FlowJo^®^ software (FlowJo, Ashland, DE, Wilmington, NC, USA). To enable the direct quantitative comparison of the cell subpopulations between the chicken lines, we acquired 10,000 live CD45^+^ cells to obtain absolute values for each sample. Data were gated according to fluorescence minus one (FMO) controls. Spleens were isolated from a total of 10 line 0 birds and from 9 line C.B12 birds, but the experiment was carried out in two rounds.

### 2.4. CD107a Degranulation Assay

Mouse anti-chicken CD107a (Table 1) was purified from hybridoma supernatant, concentrated using the mouse AbSelect TCS Purification system (Expedeon, Heidelberg, Germany) and conjugated with Lightning-link Alexa Fluor 647 (Expedeon, Germany), as per the manufacturer’s instructions. Spleen cells were isolated and resuspended at 10^6^ cells/mL in X-Vivo 15 (Lonza, Slough, UK) supplemented with 8% heat-inactivated FCS (Gibco, Paisley, UK), 2% heat-inactivated chicken serum (Gibco, UK), 100 U/mL penicillin (Gibco, UK), 100 μg/mL streptomycin (Gibco, UK) and 2 mM L-glutamine (Gibco, UK). The cells were stimulated with 200 ng/mL Ionomycin (Tochris Bioscience, Bristol, UK) and 1 μg/mL phorbol 12-myristate 13-acetate (PMA; Sigma, Gillingham, UK) in X-Vivo 15 medium for 4 h in the presence of mouse anti-chicken CD107a-AF647 (Table 1) and 1 μL/mL of BD GolgiStop (BD Bioscience, Wokingham, UK) at 37 °C and 5% CO_2_. Unstimulated cells were treated similarly but with the omission of PMA/ionomycin. After incubation, the cells were washed in FACS buffer, incubated with the appropriate antibodies and fixed as described above for flow cytometry. Spleens were isolated from line 0 and C.B12 birds (n = 10 per line), and the experiment was carried out in two rounds.

### 2.5. Western Blot

Thymus and spleen samples were collected from 12-week-old birds (n = four per line) and snap-frozen for western blot analysis. Tissue was placed in a 2 mL safe seal tube, a sterile 5 mm stainless steel bead was added and the tissue was homogenised for 2 × 2 min at 25 Hz, followed by agitation at 4 °C for 2 h in 1 mL RIPA buffer supplemented with protease and phosphatase inhibitor (TFS) using a Tissuelyser II (Qiagen, Manchester, UK). The resulting lysate was then centrifuged at 14,000 rpm at 4 °C for 20 min. The supernatants were transferred into fresh tubes, and the protein concentrations were measured by the Bradford assay (Bio-Rad, Hercules, CA, USA). Samples containing equal amounts of protein were separated under reducing conditions using SDS-PAGE, and the proteins were transferred to nitrocellulose membranes (Bio-Rad, USA). The primary antibodies used in this study were Rat anti-hCD3ε (clone CD3-12) and anti-β-actin (Table 1). Bound antibodies were detected with 1:15,000 Alexa Fluor dye conjugated secondary antibodies (LI-COR Biosciences, Lincoln, NE, USA), and the membranes were imaged with an Odyssey Fx Imaging System (LI-COR Biosciences, USA).

### 2.6. Avian Influenza Virus

LPAI virus H7N1 strain A/chicken/Italy/1067/1999 was propagated at a low multiplicity of infection (0.001) in MDCK cells for 48 h at 37 °C and 5% CO_2_ in high-glucose DMEM (Sigma, UK) supplemented with 100 U/mL penicillin, 100 μg/mL streptomycin, 2 mM L-glutamine, 0.14% BSA fraction V and 1 µg/mL TPCK-treated trypsin (Worthington Biochemical Corporation, Lakewood, NJ, USA). Mock controls were generated from culturing MDCK cells for 48 h at 37 °C and 5% CO_2_ in the absence of the virus. Viral titres were assessed by a plaque assay in MDCK cells, as previously described [15].

### 2.7. AIV Infection of Lung Macrophages

At 48 h after seeding, non-adherent cells were removed and adherent cells were infected with H7N1 at an MOI of 1 in serum-free media (1% pen/Strep/L-Glut) for 1 h at 37 °C, 5% CO_2_. The supernatant was removed, the cells were gently washed with pre-warmed PBS and 2 mL of complete RPMI-1640 (10% FCS, 1% pen/Strep/L-Glut) media was added. R848 (Resiquimod; Chemdea, Ridgewood, NJ, USA)-treated cells (5 µg/mL) were used as a positive control to measure the maximum response of each chicken line to a TLR7 agonist as a mimic for viral ssRNA, alongside untreated cells (e.g., mock). The cells (n = five samples per line and per timepoint) were harvested at 6, 24 and 48 h post-infection (hpi) via suspension in 1 mL of Trizol (TFS).

### 2.8. RNA Isolation

The cells suspended in Trizol (TFS) were harvested and lysed on a QiaShredder column (Qiagen) and centrifuged at 16,000× *g* for 3 min. The lysed cells were transferred to a 1.5 mL micro-centrifuge tube, and 0.1 mL chloroform was added to the 0.5 mL Trizol/cell lysate and mixed vigorously by pipetting. The homogenate was incubated at room temperature for 3 min and centrifuged at 12,000× *g* for 15 min for phase separation. The aqueous phase was isolated and adjusted to ~300 μL with RNase-free water, and 160 μL of RLT lysis buffer was added to the samples; 240 μL of 96–100% ethanol was added to the lysate and mixed by pipetting, and ~700 µL was transferred to an RNAeasy micro spin column (Qiagen). The samples were processed as per the manufacturer’s instructions with on-column DNase I treatment and eluted in 32 μL of RNAse-free water. RNA quality and quantity were assessed using a Nanodrop spectrophotometer ND-1000 (TFS). cDNA was synthesised from 100 ng of RNA using a High-Capacity Reverse Transcription kit (Life Technologies, Paisley, UK), according to the manufacturer’s instructions, with a random hexamer primer and oligo(dT). The cDNA was stored at −20 °C until future use.

### 2.9. Pre-Amplification of cDNA

The pre-amplification of cDNA was performed as follows: 2.5 µL of cDNA (diluted 1:5) was mixed with 5 µL of TaqMan Pre-Amp Master Mix (TFS) and 2.5 µL of a 200 nM mixed pool of primer pairs, with the following thermal protocol: 95 °C for 10 min, followed by 14 cycles of 95 °C for 15 s and 60 °C for 4 min. The pre-amplified cDNA was subsequently diluted with nuclease-free water 1:5 for the digestion of unincorporated primers with 16 U/µL Exonuclease I (*E. coli*, New England Biolabs, Ipswich, MA, USA) at 37 °C for 30 min before heat-inactivation at 80 °C for 15 min. The pre-amplified, ExoI-treated cDNA products were stored at −20 °C until use.

### 2.10. High-Throughput qPCR Using the 96.96 IFC Dynamic Array

The pre-amplified, ExoI-treated cDNA was diluted 1:5 prior to analysis through quantitative PCR (qPCR) with the microfluidic 96.96 Dynamic array Standard BioTools UK Ltd., London, UK) performed on a BioMark HD instrument (BioMark) (as described in [16]). Assay mixes were prepared by mixing 2.25 µL of 2X Assay Loading Reagent (Fluidigm), 2.5 µL of primer pair mix (1.15 µM) and 0.25 µL of low-EDTA TE buffer. Sample mixes were prepared by mixing 2.5 µL of TaqMan Gene Expression Master Mix (TFS), 0.25 µL of 20X EvaGreen DNA binding dye (Biotum), 0.25 µL of 20X GE Sample Loading reagent (Fluidigm) and 2 µL of pre-amplified, ExoI-treated cDNA (diluted 1:5). The thermal cycling conditions for qPCR were: thermal mix at 50 °C for 2 min, 70 °C for 30 min and 25 °C for 10 min, followed by a hot start step of 50 °C for 2 min, 95 °C for 10 min and then 30 cycles of 95 °C for 15 s and 60 °C for 60 s, with the fluorescence emission recorded after each cycling step. After the completion of the run, a melting curve of the amplified product was determined (60 °C for 3 s to 95 °C). Raw quantitation cycle (Cq) data were collated with the Real-Time PCR Analysis software v 3.1.3 (Fluidigm), setting the parameters of the quality threshold (0.65), baseline correction (derivative) and Cq threshold method to auto (global).

**Table 1:** Antibodies used for Flow Cytometry and Western Blotting.

Antibody	Clone	Isotype	Product Info.	Dilution
Mouse anti-chicken CD45APC	LT40	IgM	Southern Biotech	1:100 [17,18]
Mouse anti-chicken γδ TCR-FITC	TCR1	IgG1	Southern Biotech	1:500
Mouse anti-chicken αβ (Vβ1) TCR FITC	TCR2	IgG1	Southern Biotech	1:500
Mouse anti-chicken CD8β and CD8β-PE	EP42	IgG2a	Southern Biotech	1:1000 [18,19]
Mouse anti-chicken CD8α pacific blue	CT-8	IgG1	Southern Biotech	1:1000 [19,20]
Mouse anti-chicken CD4	CT-4	IgG1	Southern Biotech	1:750 [20,21]
Mouse anti-chicken CD107a *	5G10	IgG1	DSHB	1:1000
Mouse anti-chicken CD3 AF647	CT-3	IgG1	Southern Biotech	1:100
Mouse anti-chicken CD3	AV36	IgG1	UK Immunological Toolbox	1:100
Rat anti-CD3	CD3-12	IgG1	AbCam	1:1000
Rabbit anti-β actin	ab8227	polyclonal	AbCam	1:5000

* In house conjugated using Lightning-link Alexa Flour 647; DSHB-Developmental Studies Hybridoma Bank.

### 2.11. Data Processing and Analysis

The raw Cq values were processed with GenEx.v6 MultiD Analyses AB, with correction for primer efficiency and reference gene normalisation. The stability of the expression of six putative reference genes—TATA box binding protein (TBP), Tubulin alpha chain (TUBA8B), beta-actin (ACTB), beta-glucuronidase (GUSB), glyceraldehyde-3-phosphate dehydrogenase (GAPDH) and ribosomal 28S (r28S)—was evaluated via the NormFinder tool in GenEx. The geometric mean of the most stable (GAPDH, GUSB and r28S) was used to normalise all samples. Technical replicates were averaged, and the relative quantification values were assessed to the maximum Cq value obtained per gene, transformed to the logarithmic scale. Principal component analysis, assessing the overall clustering of the samples, was performed using ggplot2 in R Studio version 1.1.442.

### 2.12. Statistical Analysis

Normally distributed flow cytometry data were examined by an unpaired T-test with Welch’s correction. Data that were not normally distributed were examined by a Mann–WhitneyU test.

The AIV viral load in the lung macrophages was quantified via the 2-ΔΔCt method [17]), with the gene expression from each sample standardised using the Cq of the r28S reference gene for the same sample. Statistical analysis was performed on the log-transformed data expressed as the fold change relative to the corresponding timepoint mock control group, and differences between group means were statistically evaluated by a parametric one-way ANOVA adjusted for post hoc analysis using Tukey’s pairwise comparison.

Statistical analysis of the gene expression of lung macrophages from the IFC array was conducted to identify significantly differentially expressed genes (DEGs) between lines and between the mock and H7N1-infected or R848-stimulated groups and was performed using GenEx6, with group means compared with two-way t-tests adjusted for multiple comparisons with post hoc Bonferroni correction, with significant DEGs having a fold change >1 and <−1, illustrated in heat-maps, and the shared or unique genes annotated in Venn diagrams. For all statistical analyses, *p* values < 0.05 were considered significant. All statistical analyses were conducted using GraphPad Prism 9 or GenEx v6.

## 3. Results

A previous study by Ruiz et al. (2016) [12] using the LPAI virus infection of line C.B12 and line 0 demonstrated that line 0 birds were more resistant to infection and that the underlying mechanisms mediating greater resistance were not associated with higher adaptive immune responses, based on the levels of AIV-specific IFN-γ- and Ig-secreting cells in the spleen. Therefore, in this study, we investigated if the numbers and cytotoxic capacity of T-cell subpopulations in the spleen differed between line C.B12 and line 0. To investigate if the early responses in the respiratory tract differed between the lines, the innate immune transcriptome of macrophages isolated from the lung was analysed after in vitro infection with LPAI H7N1 or after stimulation with a TLR7 agonist (R848).

### 3.1. Line C.B12 and Line 0 Differ in γδ and αVβ_1_ T Cell Numbers

**Figure 1:**
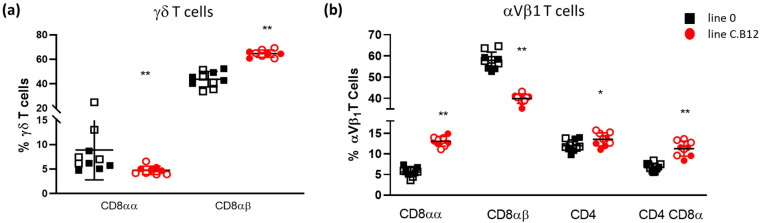
Differential γδ and αVβ1+ T cell subsets in the spleen of line 0 and line C.B21 birds. Flow cytometric analysis of cells isolated from the spleens of 4-week-old line 0 (black) and line C.B12 birds (red). To allow for the comparison of the numbers of cells between the chicken lines, 10,000 live single CD45^+^ cells were examined from each bird. (**a**) γδ T cell subsets expressing CD8αα+ or CD8αβ^+^ and (**b**) αVβ1^+^ T cell subsets expressing CD8αα, CD8αβ, CD4 and CD4^+^ CD8α^+^. * *p* < 0.05, ** *p* < 0.01. Filled symbols represent experiment 1 and open symbols represent experiment 2; n = 10 line 0 birds and n = 9 line C.B12 birds. Black bar represents the mean. Error bars represent the standard deviation.

Using flow cytometric analysis, cell subpopulations were quantified in the spleen of 4-week-old birds, and to enable the quantification of the proportion of cells between the chicken lines, 10,000 live single CD45^+^ cells were collected per sample. The gating strategies are shown in Supplementary Figures S1 and S2. First, the γδ T cells (TCR1^+^) and αVβ_1_ T cells (TCR2^+^) were assessed. Significantly lower percentages of CD8αα^+^ γδ T cells were present in the spleen of C.B12 birds compared to those in line 0 birds, whereas significantly higher percentages of CD8αβ^+^ γδ T cells were detected in the spleens of the C.B12 birds (*p* < 0.01; Figure 1a).

The percentage of αVβ_1_ T cell subsets also differed between the lines. Susceptible line C.B12 had a significantly higher percentage of CD8αα^+^ αVβ_1_ T cells, with a conversely significantly lower percentage of CD8αβ^+^ αVβ_1_ T cells compared to line 0 (*p* < 0.01; Figure 1b). The percentage of CD4^+^ αVβ_1_ T cells was significantly higher in the C.B12 birds (*p* < 0.05), although the difference was small and the biological relevance was questionable. Moreover, a larger difference was found in the percentage of CD4^+^CD8α^+^ double-positive αVβ1 T cells, which was significantly higher in the C.B12 birds compared with the line 0 chickens (*p* < 0.01; Figure 1b). The data were highly reproducible, and no differences were found between the two experiments. In summary, line C.B12 birds had a higher proportion of conventional γδ T cells and unconventional αVβ1 T cells in the spleen.

### 3.2. Phenotypic Analysis of Splenic Cytotoxic Cells

**Figure 2:**
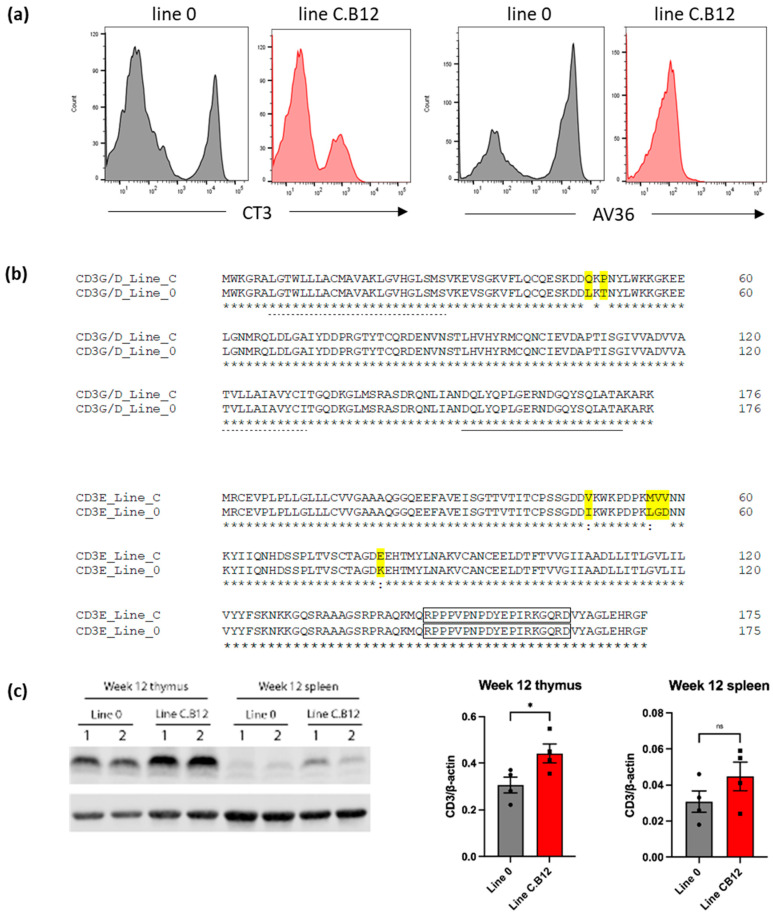
Polymorphisms in CD3 of line 0 and line C.B12 birds. (**a**) Flow cytometric analysis of splenocytes of 4-week-old line 0 (black) and line C.B12 birds (red), stained with mouse anti-chicken CD3 clone CT3 or mouse anti-chicken CD3 clone AV36. Cells were gated on 10,000 live, single CD45^+^ cells. Representative of five birds. (**b**) Comparison of the CD3 chains between line C and line 0. Dotted lines represent the transmembrane domain, and the underline represents an ITAM domain. Differences in the amino acid sequence between the lines are highlighted in yellow. The boxed area is the epitope recognised by the CD3-12 antibody. (**c**) Representative images of western blot analyses for lymphocytes from the lung, thymus and spleen of two 12-week-old line 0 and line C.B12 birds using the anti-CD3ε clone CD3-12 (**left** panel). Quantitative analyses for CD3 levels relative to the actin from thymus (**middle** panel) and spleen cells (**right** panel) are shown. Data points are for cells collected from individual birds (n = four per line). * *p* < 0.05.

To characterise the differences in splenic cytotoxic cell populations, NK cells and cytotoxic T lymphocytes (CTLs), further flow cytometric analyses were performed. While NK cells express intracellular CD3, they do not express this molecule on the cell surface, whereas CTLs express surface CD3 [22]. Therefore, these cytotoxic cells can be distinguished by flow cytometry based on a gating strategy for surface CD3 expression. We discovered that anti-CD3 clone CT3 IgG bound to a lower level on C.B12 splenocytes compared to line 0 cells, while anti-CD3 clone AV36 bound well to line 0 splenocytes but did not detectably bind to C.B12 T cells (Figure 2a). The sequence comparison of the CD3ε and the CD3γ/δ genes of line 0 and line C.B12 birds showed multiple changes in the amino acid sequence in the extracellular domains of both chains (Figure 2b), which may explain the differential binding properties of clone CT3 and AV36 antibodies. Whatever the reason for the differential recognition of CD3 by the two reagents, it made it impossible to identify the NK cell populations on the basis of the absence of CD3 surface expression.

To determine if the chicken lines expressed different levels of CD3 protein, expression was analysed by western blot following the lysis of thymus and spleen cells and the detection of CD3 using a cross-reactive clone CD3-12 antibody that recognises a cytoplasmic epitope of CD3ε which is identical in line 0 and line C.B12 (Figure 2b). The total CD3 protein levels in the thymus and spleen of C.B12 birds were higher than those in line 0 birds (Figure 2c, left panel), but on the quantification of the replicate samples, the difference was only statistically significant in thymus (*p* < 0.05) (Figure 2c, middle and right). The absolute number of CD3-expressing cells in the spleen was not determined, and therefore, the differences could be due to the difference in the number of cells. However, as the majority of the cells in the thymus are T cells and NK cells, the data suggest that, although the surface expression of CD3 may be lower in C.B12 birds, the amount of intracellular CD3 tends to be higher.

Since gating in the absence or presence of CD3 to distinguish NK cells from CTLs was not feasible due to polymorphisms in CD3, as well as its differential expression across the lines, only the degranulation of T cell subsets was further investigated.

### 3.3. Differential Degranulation Potential of γδ and αVβ_1_ T Cells

**Figure 3:**
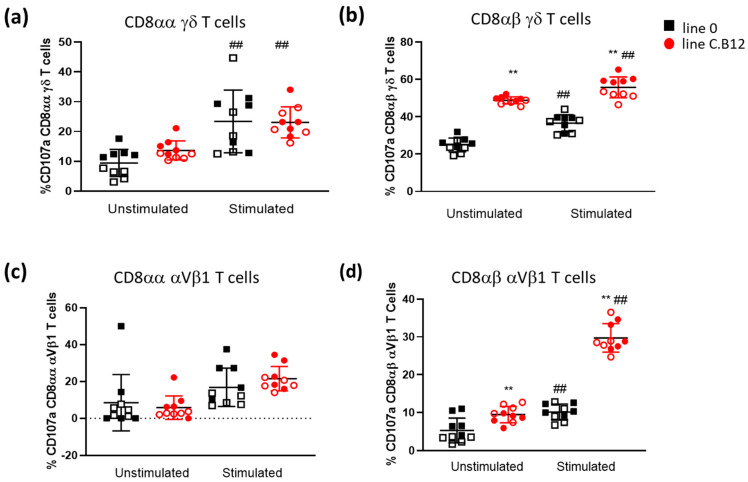
CD107a degranulation assay of γδ T and αVβ1^+^ T cell subsets in the spleen of line 0 and line C.B12 birds. Flow cytometric analysis of surface CD107a on naïve and stimulated γδ T cells (**a**,**b**) and αVβ1^+^ T cells (**c**,**d**) isolated from the spleens of 4-week-old line 0 (black) and line C.B12 (red) birds. CD8αα^+^ populations (**a**,**c**) and CD8αβ^+^ populations (**b**,**d**). ** *p* < 0.01 between lines, ## *p* < 0.01 within lines. Filled symbols represent experiment 1 and open symbols represent experiment 2; n = 10 birds per line. Black bar represents the mean. Error bars represent the standard deviation.

After finding the difference in γδ and αVβ_1_ T cell subpopulations in the spleens of line C.B12 and line 0 birds, we assessed their cytotoxic potential using a CD107a degranulation assay with unstimulated cells and in vitro stimulated cells. The CD107a^+^ CD8αα^+^ γδ T cell percentage was not significantly different between the chicken lines in both the unstimulated and PMA/ionomycin-stimulated cells (Figure 3a). In contrast, the percentage of CD107a^+^ CD8αβ^+^ γδ T cells was significantly higher in both the unstimulated and stimulated cells isolated from the C.B12 birds, compared with the percentage of CD107a^+^ cells in the unstimulated and stimulated population isolated from the line 0 birds (*p* < 0.01; Figure 3b).

Similar to the CD8αα^+^ γδ T cells, the CD107a expression in CD8αα^+^ αVβ_1_ T cells did not differ significantly (Figure 3c) following PMA/ionomycin stimulation. In contrast, line C.B12 had a higher expression of CD107a in the unstimulated CD8αβ^+^ αVβ_1_ T cells (Figure 3d). After stimulation, CD107a expression increased significantly in the CD8αβ^+^ αVβ_1_ T cells isolated from both line C.B12 and line 0, but the increase was significantly higher in the cell population isolated from C.B12 compared to that isolated from line 0 (*p* < 0.01; Figure 3d). In summary, in addition to the higher numbers of CD8αβ^+^ γδ T cells and CD4^+^CD8αα^+^ αVβ_1_ T cells, the more susceptible C.B12 line has a significantly higher percentage of CD8αβ^+^ T cells expressing CD107a both directly ex vivo and post-stimulation in vitro. This suggests a continuous higher state of the degranulation of its cytotoxic cells, which may be associated with increased sensitivity and immunopathology.

### 3.4. Differential Innate Immune Responses in Lung Macrophages

**Figure 4:**
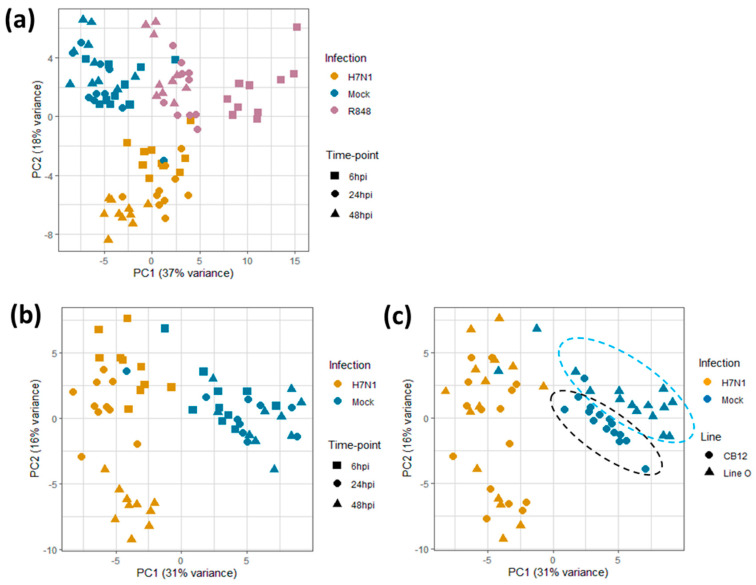
Principal component analysis (PCA) of the global gene expression of viral innate immune-related genes in lung macrophages using Fluidigm qPCR array. Lung macrophages were isolated from line 0 and line C.B12 and in vitro-infected or stimulated for 6, 24 or 48 h (n = five birds per line; three-way split over treatments and timepoints). (**a**) PCA plot of collated data from line 0 and line C.B12 showing clustering of the samples in terms of infection/stimulus: H7N1-infected (orange), mock (blue) and R848-stimulated (pink). (**b**,**c**) PCA plots of lung macrophages infected with H7N1 (orange) or mock (blue) indicate clusters and transcriptional differences in gene expression related to the (**b**) timepoint and (**c**) genetic line, with the C.B12 cluster highlighted in a black-dashed ellipse and the line 0 cluster highlighted in a blue-dashed ellipse.

To investigate if the differential responses to AIV may be caused by a hereditary difference in the ability of the lines to initiate an immune response, we investigated the innate immune gene expression in mock lung macrophages and in response to an ssRNA viral mimic, the TLR7 agonist R848, using an RT-qPCR Fluidigm array containing 89 genes (82 innate immune genes, AIV M gene, 6 reference genes and 1 non template control; Supplementary Table S1). First, to explore the degree of heterogeneity in the transcriptional profiles, the data were compared using principal component analysis. PCA was performed using all of the gene expression data for the R848-stimulated, H7N1-infected and mock (i.e., untreated) lung macrophages from both lines and at all timepoints, and this showed that the response of the macrophages clustered in three distinct groups, in terms of their infection/treatment status (Figure 4a). Further analysis suggested only small differences in the kinetics of the response to H7N1 infection at 48 hpi compared to 6 and 24 hpi (Figure 4b), but this was not attributable to the underlying genetic differences between the lines. The comparison of the gene expression of the mock-infected macrophages showed clustering of the data into the two genetic lines (Figure 4c), suggesting a contribution of the genetic background of the birds.

**Figure 5:**
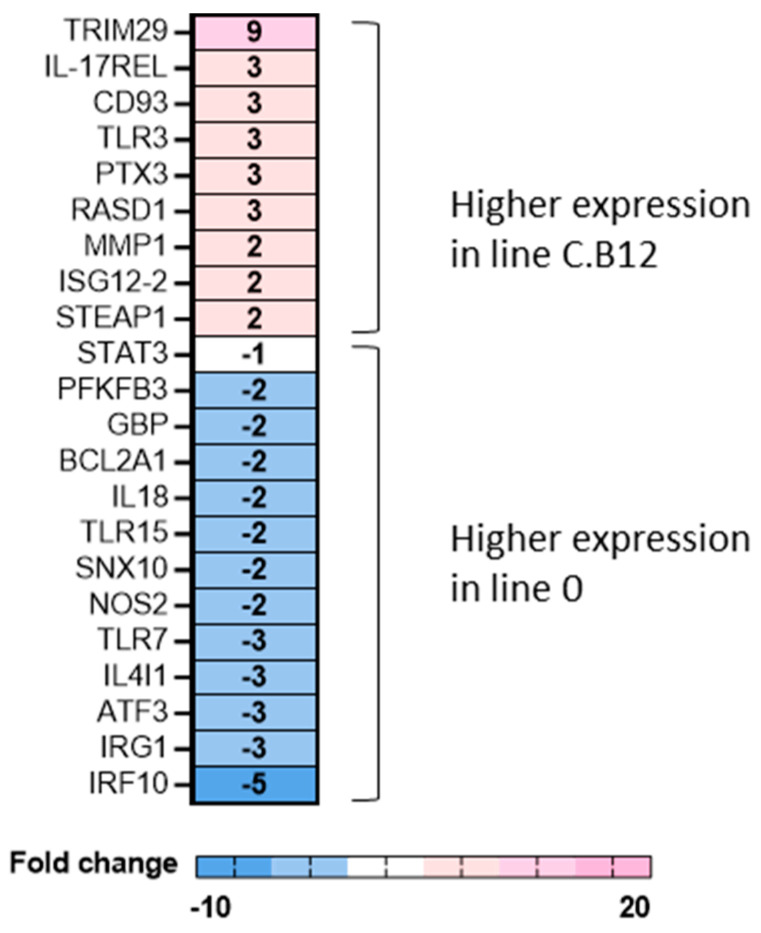
Hereditary differences in innate immune gene expression in lung macrophages from line C.B12 and line 0 chickens. Significant DEGs were identified by comparing the relative expression values, calculated through comparison to the maximum Cq for individual genes for lung macrophages from mock/untreated line C.B12 and line 0 chickens, with a significance level set at *p* < 0.05; fold change >1 (n = five per line). Heat map illustrates the mean fold change associated with significant DEGs between line C.B12 and line 0, and the fold change values are specific to line C.B12 (i.e., C.B12 v line 0). Fold change values are represented on a divergent, intensity colour scale showing the expression of upregulated genes (pink) to downregulated genes (blue).

To further investigate this difference between the lines in the mock-infected macrophages, we compared the expression of 82 innate immune genes. The lung macrophages of line C.B12 and line 0 were compared, and the untreated cells at 6 h indicated nine genes enriched in line C.B12 and 12 genes enriched in untreated line 0 lung macrophages (i.e., downregulated compared to C.B12 macrophages; Figure 5). The genes more highly expressed in line C.B12 included *TRIM29*, a negative regulator of innate immune responses, including in alveolar macrophages [23], *IL17REL,* a soluble IL-17R predicted to be a negative regulator of IL-17 [24], and *CD93,* a C-type lectin that is expressed in activated macrophages, among other things [24]. The lung macrophages isolated from line 0 expressed higher mRNA levels of interferon-regulated genes compared to line C.B12, including *IRF10*, which is involved in the upregulation of two primary IFN-γ target genes (MHC I and guanylate-binding protein, *GBP*, also upregulated in line 0 macrophages) and resembles the function of mammalian IRF1 [25]. In mouse and human macrophages, IRF1 is described as a transcriptional regulator of immune responsive gene 1 (*IRG1*) [26], which, in turn, has an antiviral role and was significantly upregulated in line 0 lung macrophages. Also upregulated in line 0 cells was *ATF3,* a key regulator of macrophage IFN responses in mice which limits the inflammatory response by controlling the expression of a number of cytokines and chemokines [27]. While dsRNA-binding *TLR3* showed a threefold increase in line C.B12, the expression of ssRNA-binding *TLR7* was threefold higher in line 0.

**Figure 6:**
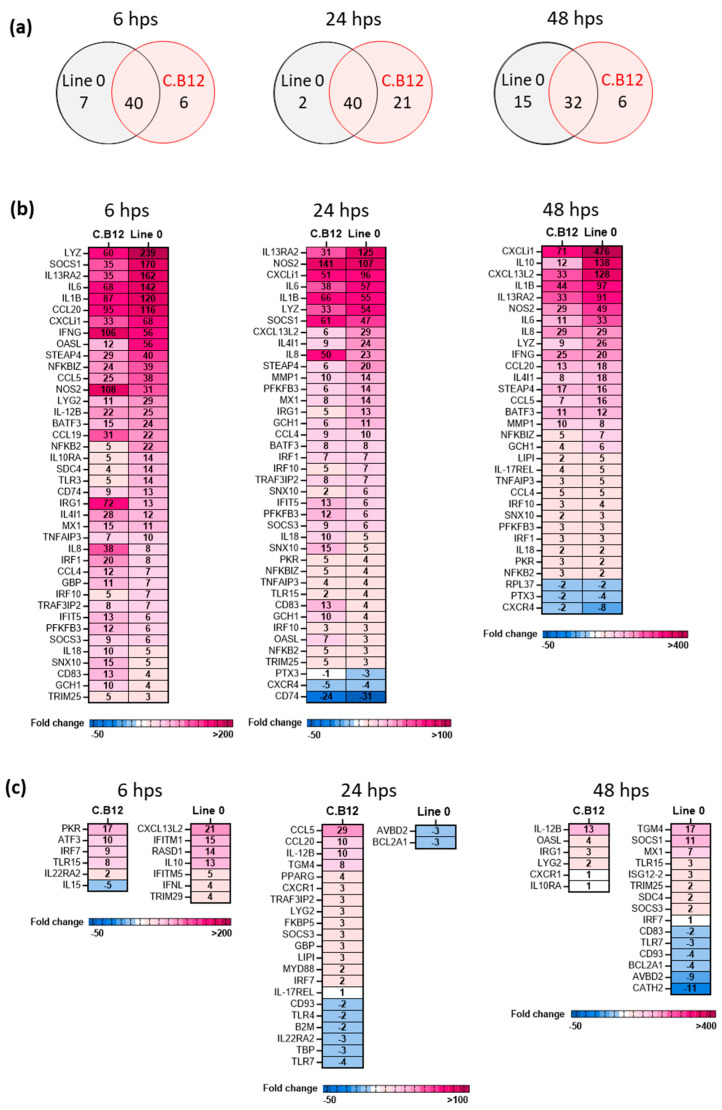
R848 stimulation of lung macrophages induces a maximal immune response with kinetic differences across genetic lines. Significant DEGs were identified by comparing the relative expression values for R848-stimulated lung macrophages to their corresponding mock control, which was performed intra-line (i.e., C.B12 R848-stimulated v C.B12 mock) and individually per timepoint, with a significance level set at *p* < 0.05; fold change >1 (n = five/line/timepoint). (**a**) Venn diagrams presenting the number of significant DEGs that are shared among or are specific to line 0 and line C.B12 in response to R848 stimulation, split by timepoint. Heat maps illustrating the fold change associated with: (**b**) significant DEGs shared among line 0 and line C.B12 and (**c**) unique to a specific line at 6 hps, 24 hps and 48 hps. Fold change values are represented by a divergent intensity of colour.

Next, we compared the maximal inducible response in the lung macrophages isolated from both chicken lines to ssRNA as a mimic for AIV infection. Here, the lung macrophages were stimulated with R848 and analysed at 6, 24 and 48 h post-stimulation (hps). The number of shared and unique significant DEGs over time indicated that a rapid and high induction of innate immune genes compared to the line-matched controls was detected in both lines at 6 hps (Figure 6a). However, the magnitude of the response to R848-stimulation was greater in line 0 compared to that in line C.B12 (Figure 6b). Of the shared DEGs, six significant DEGs were upregulated with a fold change (FC) >100 (*IL1B*, *CCL20*, *SOCS1*, *LYZ*, *IL13RA2*, *IL-6*) in line 0, whereas only two significant DEGs were upregulated >100-fold in line C.B12 (*NOS2*, *IFNG*). Except for *NOS2* (inducible NOS) in line C.B12, the FC of the extremely highly upregulated genes decreased at 24 hps. Although TLR ligands induce rapid changes in innate gene expression, the effects were still measurable at 48 hps, with a very high expression of *CXCLi1*, *IL10*, *CXCL13L2*, *IL13RA2* and *IL1B* in line 0 (FC > 90) and line C.B12 (FC > 30, except for *IL10*).

Similar to the shared significant DEGs, the unique DEGs at 6 hps were expressed at a higher level in line 0, with an FC > 10 for *IL10*, *RASD1*, *chIFITM1* and *CXCL13L2*, whereas in line C.B12, only *PKR* had an FC > 10 (Figure 6c). At 24 hps, line 0 only had two unique DEGs (*AVBD2*, *BCL2A1*; FC -3), whereas C.B12 had 21 unique DEGs, with *CCL5*, *CCL20* and *IL12B* showing an FC ≥ 10. At 48 hps, the *IL12B* expression in C.B12 birds was still highly upregulated (FC 13), with no unique gene downregulated, whereas line 0 had eight genes up- and six genes downregulated, with an FC ≥2 or ≤2, respectively. *AVBD2* and *CATH2* were strongly downregulated and *TGM4* and *SOCS1* were highly upregulated in line 0 at 48 hps. In conclusion, the resistant line 0 macrophages induced a more rapid and stronger antiviral response after stimulation with a TLR7 agonist than the cells from C.B12 birds [28].

#### Line-Specific Responses to H7N1 Infection

The underlying genetic influence on the response to AIV infection was investigated by comparing the transcriptomic response of lung macrophages from line 0 and line C.B12 chickens infected with LPAI H7N1. First, we analysed the viral load and found significantly higher levels of AIV RNA at 6 hpi in infected cells (MOI = 1) from line 0 compared to those from line C.B12 (*p* < 0.001). However, no significant differences in the levels of AIV viral RNA were found when comparisons were made between lines at 24 or 48 hpi, nor between the 6 h and 24 h post-infection timepoints (Supplementary Figure S3), suggesting that the differences in the innate immune responses between the lines in this study were not due to a difference in the viral load.

**Figure 7:**
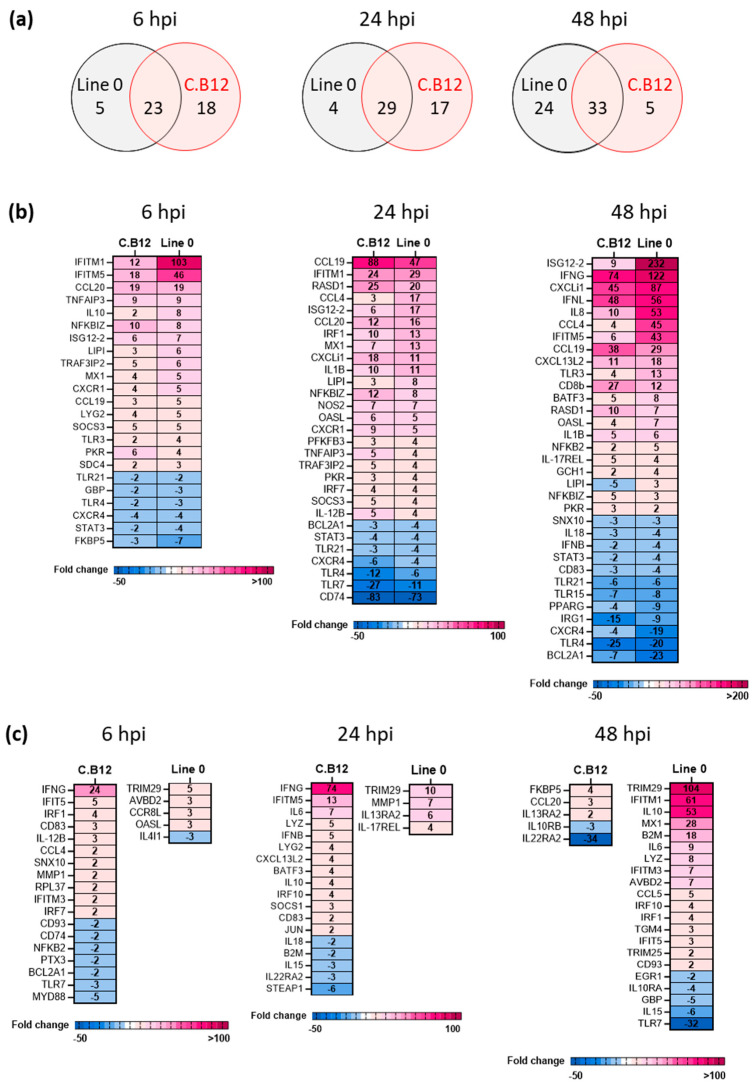
H7N1 infection of lung macrophages shows differences in the immune response kinetics and intensity between susceptible and resistant lines. Significant DEGs were identified by comparing the relative expression values for H7N1-infected lung macrophages to their corresponding mock control, and this was performed intra-line (i.e., C.B12 infected v C.B12 mock) and individually per timepoint, with a significance level set at *p* < 0.05; fold change >1 (n = five/line/time-point). (**a**) Venn diagrams presenting unique and shared numbers of significant DEGs of line 0 and line C.B12 in response to H7N1. (**b**) Heatmaps illustrating the fold change associated with significant DEGs that are shared among line 0 and line C.B12 and are (**c**) unique to a specific line at 6 hpi, 24 hpi and 48 hpi. Fold change values are represented on a divergent, intensity colour scale showing the expression of upregulated genes (pink) to downregulated genes (blue).

At 6 hpi, 46 significant DEGs were identified, with 23/46 shared among H7N1-infected lung macrophages from line C.B12 and line 0, while 18/46 and 5/46 were unique to lines C.B12 and 0, respectively (Figure 7a). At 24 hpi, 29/50 of the significant DEGs were shared among the H7N1-infected lung macrophages from line C.B12 and line 0, with 17/50 and 4/50 unique to line C.B12 and line 0 responses to infection, respectively. At 48 hpi, 33/62 significant DEGs were shared between the lines, but in contrast to the earlier time-points, a larger number of genes were uniquely expressed in H7N1-infected lung macrophages from line 0: 24/62 versus 5/62 in line C.B12 compared to their respective controls (Figure 7a).

Differences in the innate immune responses were found with regard to the kinetics and intensity of the response, and a rapid but different antiviral response was found when comparing the lines. Although many similar genes were up- or downregulated simultaneously in both lines compared to their respective mock control (Figure 7b), at 6 hpi, the interferon-induced transmembrane proteins *IFITM5* and *chIFITM1* mRNA expression increased by 46- and 103-fold, respectively, in the resistant line 0 compared to 18- and 12-fold increases in C.B12 cells. At 48 hpi, many genes were upregulated in both lines in response to LPAI, but to a higher extent in line 0, especially the chemokines *CCL4* (MIP1β), *IL8* (CXCLi2) and *CXCLi1* (K60) and the ISGs and IFNs *ISG12-2*, *IFITM5*, *IFNL* and *IFNG*.

The DEGs that were uniquely regulated within each line are shown in Figure 7c. The more susceptible line C.B12 reacted swiftly at 6 and 24 hpi, with a larger number of uniquely expressed genes and also more downregulated DEGs. The downregulated genes included *MYD88* and *TLR7*, whereas *IFNG* was uniquely upregulated in C.B12 at 6 hpi and increased by 74-fold at 24 hpi. In contrast, in line 0, a significant increase in *IFNG* was only found at 48 hpi (122-fold). At 48 hpi, line 0 uniquely upregulated more antiviral genes, including *chIFITM1*, *chIFITM3*, *IFIT5* and *MX1*. However, the highest upregulated genes were the negative regulators of the innate immune responses *TRIM29* and *IL10*. This may suggest that, in line 0, besides a strong antiviral response, a vigorous negative feedback loop is established to regulate the pro-inflammatory and innate anti-viral immune responses and prevent immunopathology.

## 4. Discussion

The resistance and susceptibility to diseases including AIV in chickens have been associated with the particular genetic background of the birds [29]. Therefore, genetically defined lines provide a useful tool for investigating the mechanisms underlying the differential resistance to AIV. In this study, we used two chicken MHC I congenic lines: line C.B12 (haplotype B12) and line 0 (haplotype B21), which are, respectively, more susceptible and more resistant lines, as defined by the substantial differences in the viral shedding trajectories [12]. The resistance to infection appeared to be less dependent on the adaptive immune responses in these lines, based on the expansion of AIV-specific IFN-γ-secreting cells or the production of influenza-specific antibodies by splenocytes. In a further attempt to elucidate the mechanisms underlying resistance, we focused on analysing the differences between the lines through the quantification of different immune cell subsets in the spleen and their associated degranulation ability as a proxy of cytotoxic potential and by a comparative analysis of the innate immune gene expression in macrophages isolated from the respiratory tract post-infection with AIV.

In our study, differences in conventional and unconventional γδ and αβ T cell subsets were identified in the spleens of naïve chickens. In contrast to the highly polymorphic nature of the MHC class I and class II complexes that restrict αβ T cell responses, the vast majority of γδ T cell ligands reported so far are non-polymorphic in nature (reviewed in [30]), suggesting a role for γδ T cells in the innate immune system sensing molecular signals of microbial and non-microbial stress. Moreover, mammalian γδ T cells can be primed independently of the TCR [31]. Here, we show that the susceptible line C.B12 has a significantly higher number of CD8αβ γδ T cells and CD8αα αVβ1 T cells. Chickens, compared to humans and mice, have higher frequencies of γδ T cells, representing 20–50% of the T cell population [32,33]. Chicken γδ T cells can secrete both pro- and anti-inflammatory cytokines including IFN-γ [34], IL-17A [35], IL-6, IL-10 and IL-13 [36]. They also express TLR3 and TLR4 [34]. In addition, Fenzl et al. [37] reported that a large proportion (up to 50%) of chicken splenic γδ T cells exhibited spontaneous cytotoxicity, and this effect could be enhanced by IL-2 and IL-12 supplementation. In our study, splenic CD8αβ γδ T cells in the line C.B12 birds, but not in the line 0 birds, also showed ~50% spontaneous degranulation. Interestingly, the lung macrophages isolated from line C.B12 and stimulated with R848 had a consistently high expression of *IL12B* over time, whereas *IL12B* was only upregulated at 6 hps in line 0. Thus, the innate function of the γδ T cells may resolve part of the disparity in the previously discounted role for the adaptive immune response in the differential susceptibility of line C.B12 and line 0 birds.

The CD8 molecule is expressed as either an αα homodimer or an αβ heterodimer on cytotoxic T cells and functions as a co-receptor, with the TCR binding to the MHC I/peptide complex. CD8αα and CD8αβ are not functional homologues, with the CD8αα homodimer being a weaker co-receptor for MHC class I, and, in fact, it is described as a co-repressor in mammalian studies [38,39]. In addition, the CD8αα homodimer also binds to non-classical MHC I in mice [40]. The function of CD8αα T cells remains contentious, even in mammals, and requires further investigation, but in the context of our findings in the chicken lines, the increased CD8αα αVβ_1_ T cell population and the CD4 CD8α double-positive population in the susceptible line C.B12 could potentially validate a repressive role for the CD8αα homodimer. Further functional studies of these subsets and TCR repertoires are essential in understanding the role of chicken non-conventional T cells in immune surveillance, host–pathogen interaction and the regulation of innate and adaptive responses.

To investigate if lung macrophages, which are part of the first line of respiratory pathogen defence, differed between the chicken lines, we determined innate immune gene expression in untreated birds and after stimulation with a TLR7 agonist (R848) and infection with H7N1 LPAI in vitro. The lack of increased viral RNA over time suggests that the virus is taken up or enters the macrophages, but the cells do not support extensive virus replication. Similarly, others have reported that the infection of primary splenic macrophages supported the synthesis of the viral protein, but infectious virus was not produced [41]. The chicken monocyte/macrophage cell lines HTC [42] and HD11 [43,44] are both susceptible to AIV, although differences between the virus strains are described [44]. Macrophages isolated from the lungs of influenza-infected mice and cultured ex vivo to determine if they might be productively infected in vivo did not produce infectious seasonal influenza virus but did support the replication of the pandemic 1918 virus [45]. In conclusion, lung macrophages can be infected with LPAI strains but likely do not support the high production of virus particles, which is the reason for not exploring the responses to other AIV strains in this study.

The analysis of a set of innate immune genes in primary macrophages isolated from the lung suggests that the line 0 macrophages express higher levels of antiviral genes, whereas line C.B12 macrophages express higher levels of negative regulator genes. Stimulation with R848 induced a strong innate response in both lines, with, in general, a stronger response in the more resistant line 0. A major difference was the induction of a type II IFN response, with a very high expression in line C.B12 and a rapid induction at 6 h after stimulation with R848. The induction of *IFNG* in line 0 surpassed that of C.B12 but at a much later timepoint, 48 hpi. The *IFNG* expression in response to H7N1 followed a similar pattern in that the kinetics of the expression differed, although both lines upregulated this gene to a very high extent. Interestingly, with the more susceptible line C.B12 also having a significantly higher proportion of non-conventional CD8αα T cells, known to secrete IFN-γ [46] and higher spontaneous degranulation, these birds may be prone to overreact to stimuli or viruses.

After infection with H7N1 AIV, *IFITM1* was the most upregulated gene at 6 hpi in resistant line 0. The overexpression of this gene in vitro has been shown to increase the resistance of avian cells to AIV infection [47], probably by a block in membrane fusion, which is crucial to the entry and further replication of this virus. In ducks, *IFITM1* was highly upregulated after infection with HPAI H5N1, whereas little response was seen in chickens [8], further suggesting it is a contributory factor to resistance. *ISG12-2* was stably expressed over time in line C.B12 (FC 6-9), whereas it was the highest DEG in line 0 at 48 hpi (FC = 232). We previously showed the upregulation of this gene in AIV-infected chicken lung and tracheal organ cultures [48], and it was identified as a potential gene involved in the resistance to infectious bronchitis virus (IBV) cultures [48]. More recently, the overexpression of ISG12-2 was shown to inhibit Newcastle Disease virus (NDV) replication, and in vivo vaccination with a recombinant NDV-expressing ISG12-2 improved the protection against a virulent challenge [49]. ISG12-2 will be an interesting candidate gene for further mechanistic studies.

Another difference between the lines was the unique higher expression of immune regulatory genes at 48 hpi in the more resistant birds, including *IL10* and *TRIM29*, suggesting that, besides a high induction of antiviral genes, a regulatory pathway is induced alongside the pro-inflammatory responses. Cells of the innate immune system such as macrophages, NK cells and granulocytes can play a beneficial role in the response to influenza by killing and clearing virus-infected cells and secreting immune mediators to facilitate more of a recruitment of inflammatory cells. However, this recruitment can lead to an imbalance between antiviral and inflammatory responses and may contribute to different outcomes of infections in these chicken lines, as well as between differentially susceptible bird species, i.e., chickens and ducks. Hereditary differences in uninfected birds and differences in the kinetics of the cytokine and chemokine responses in MHC I inbred chicken lines have been reported previously after infection with IBV [50], infectious bursal disease virus [51], *Eimeria maxima* [52] and *Campylobacter jejuni* [53], as well as in vitro after the infection of macrophages with Marek’s disease virus [54]. Recently, the transcriptomic analysis of tracheal tissue from chickens infected with four distinct LP H7 viruses, characterised by different histories of pathogenicity evolution in the field, highlighted that the first line of defence against AIVs was initiated with a different magnitude to those viruses that switched from an LP to an HP genotype and phenotype in the field [55]. Therefore, not only the identification of genes involved in the resistance against viral infections but also the magnitude and kinetics of the responses should be taken into account, as many genes are differentially regulated in susceptible and resistant birds.

## 5. Conclusions

The overall evidence in our study showed that the more susceptible line C.B12 birds had a significantly higher number of non-conventional CD8αα αVβ1 and CD4 CD8α double-positive αVβ1 T cells, a lower threshold of activation of cytolytic degranulation in conventional CD8αβ γδ T cells and CD8αβ αVβ1 T cells and a lower expression of immune regulatory genes and a higher type II IFN response after the infection of lung macrophages with H7N1. Together, these findings suggest a potential role of immunopathology in mediating susceptibility in C.B12 birds. Although both lines strongly upregulated antiviral genes, the kinetics and magnitude of the responses were significantly different, which likely contributes to conferring resistance to LPAI. This study therefore indicates that immunopathology warrants further investigation into the role of the dissemination of and susceptibility to AIV. In identifying differential hereditary responses associated with resistance to AIV, this study also highlights the potential to breed for more robust poultry to improve biosecurity in the context of the global threat posed by Influenza A virus.

## Data Availability

Not applicable.

## Supplementary Materials

The following supporting information can be downloaded at: https://www.mdpi.com/article/10.3390/v15030591/s1: Figure S1: Gating strategy for establishing CD45-positive cells. Figure S2: Gating strategy for establishing T cell subsets. Figure S3: AIV viral RNA load in lung macrophages from line 0 and line C.B12. Figure S4: Representative example of a flow cytometric analysis of lung macrophages showing the purity of the adherent cells using a gating strategy based on live cells and staining for CD45 (clone LT40) and CSF1R (clone ROS-AV170). Table S1: Innate immune genes on the Fluidigm 96.96 IFC Dynamic Array.
