# Supplementary material for: Comparative Analysis of Different Inbred Chicken Lines Highlights How a Hereditary Inflammatory State Affects Susceptibility to Avian Influenza Virus
Source: Viruses. 2023 Feb 21;15(3):591. doi: 10.3390/v15030591 (PMC10052641; doi:10.3390/v15030591)
Supplement: Supplementary file 1 [file viruses-15-00591-s001.zip › Supplementary Figure S1.pptx]

## Slide 1
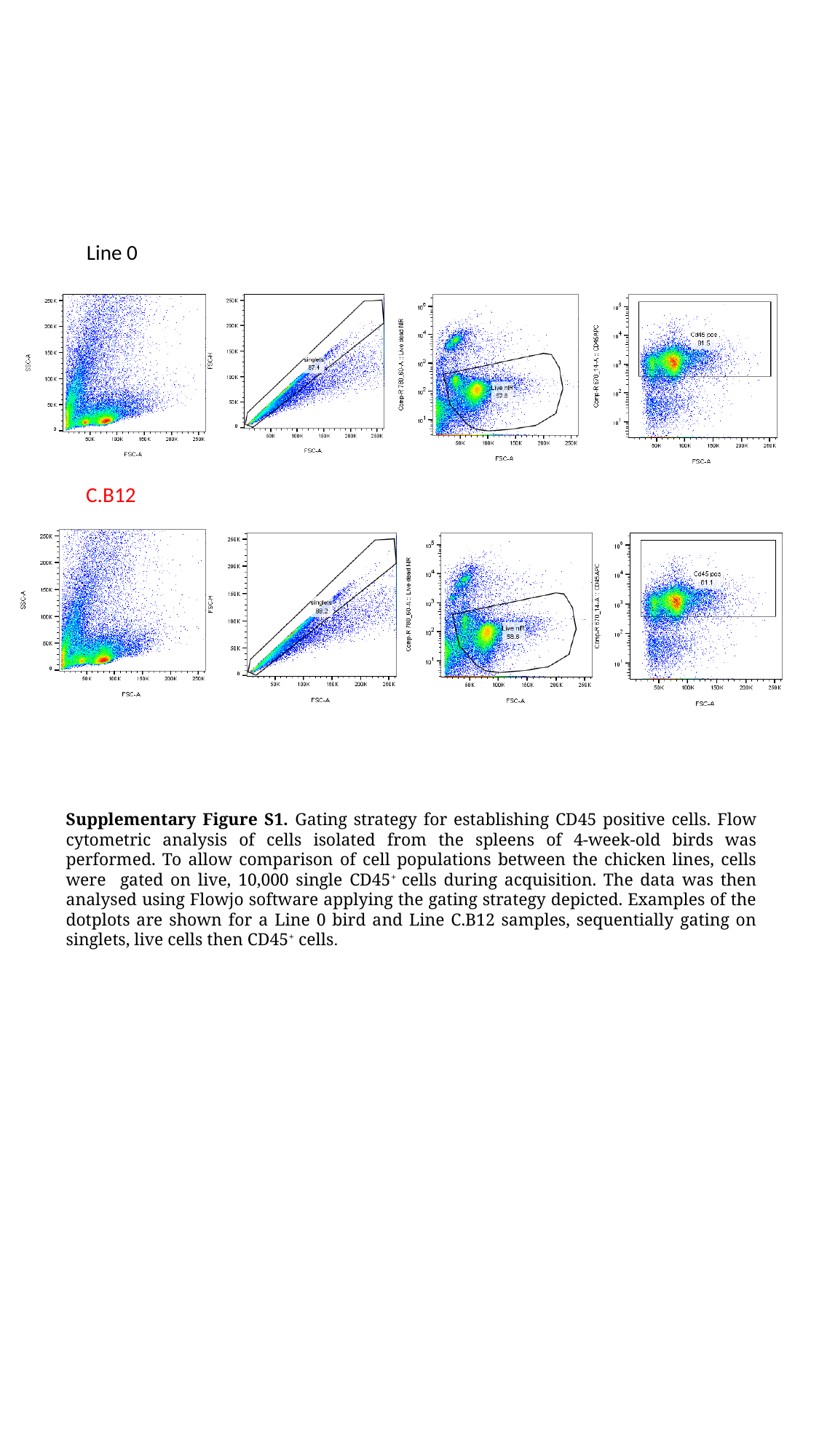

Line 0
C.B12
Supplementary Figure S1. Gating strategy for establishing CD45 positive cells. Flow cytometric analysis of cells isolated from the spleens of 4-week-old birds was performed. To allow comparison of cell populations between the chicken lines, cells were gated on live, 10,000 single CD45+ cells during acquisition. The data was then analysed using Flowjo software applying the gating strategy depicted. Examples of the dotplots are shown for a Line 0 bird and Line C.B12 samples, sequentially gating on singlets, live cells then CD45+ cells.
