# Supplementary material for: Comparative Analysis of Different Inbred Chicken Lines Highlights How a Hereditary Inflammatory State Affects Susceptibility to Avian Influenza Virus
Source: Viruses. 2023 Feb 21;15(3):591. doi: 10.3390/v15030591 (PMC10052641; doi:10.3390/v15030591)
Supplement: Supplementary file 1 [file viruses-15-00591-s001.zip › Supplementary Figure S2c-d.pptx]

## Slide 1
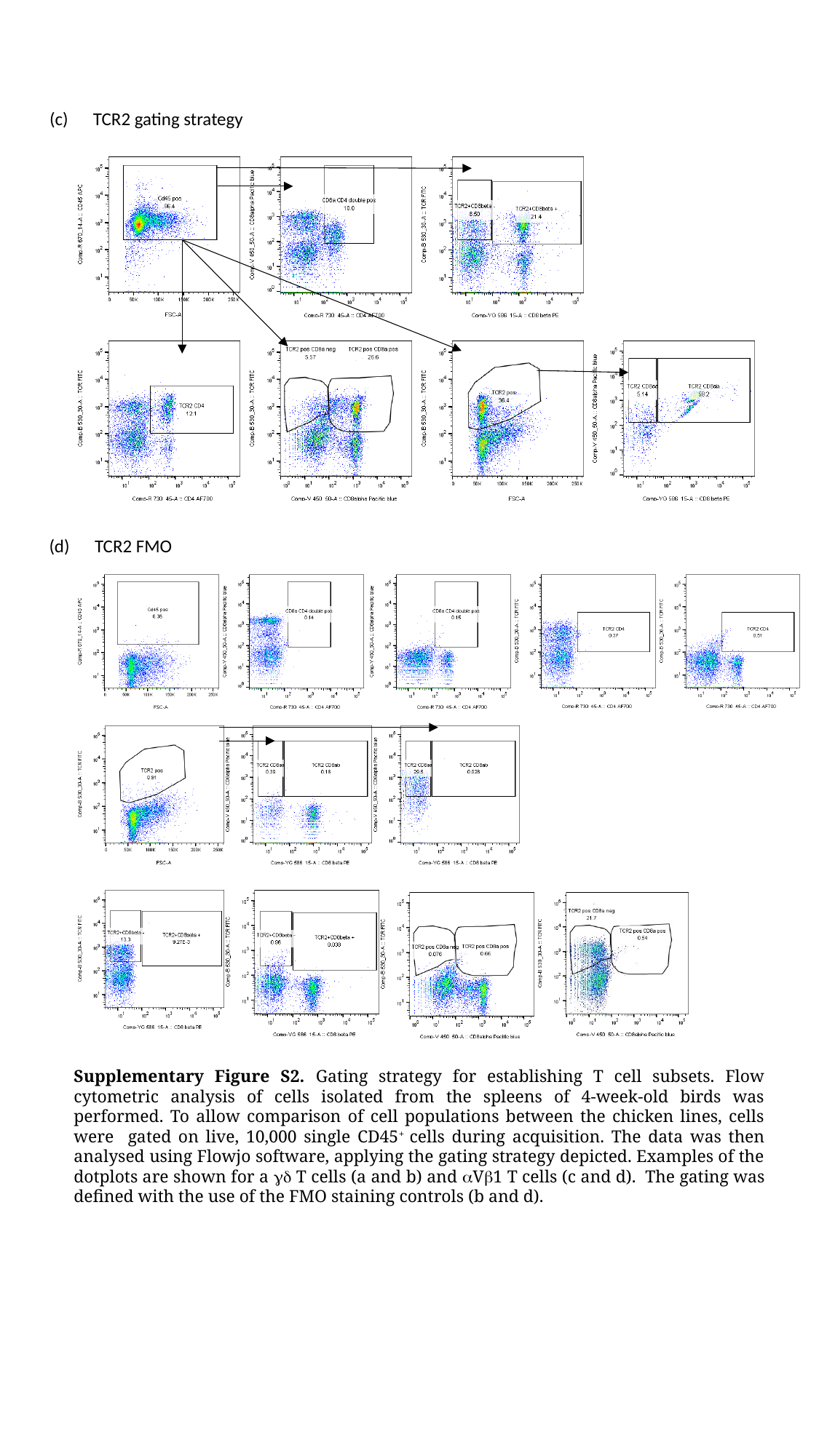

(c) TCR2 gating strategy
(d) TCR2 FMO
Supplementary Figure S2. Gating strategy for establishing T cell subsets. Flow cytometric analysis of cells isolated from the spleens of 4-week-old birds was performed. To allow comparison of cell populations between the chicken lines, cells were gated on live, 10,000 single CD45+ cells during acquisition. The data was then analysed using Flowjo software, applying the gating strategy depicted. Examples of the dotplots are shown for a  T cells (a and b) and αVβ1 T cells (c and d). The gating was defined with the use of the FMO staining controls (b and d).
