# Supplementary material for: Comparative Analysis of Different Inbred Chicken Lines Highlights How a Hereditary Inflammatory State Affects Susceptibility to Avian Influenza Virus
Source: Viruses. 2023 Feb 21;15(3):591. doi: 10.3390/v15030591 (PMC10052641; doi:10.3390/v15030591)
Supplement: Supplementary file 1 [file viruses-15-00591-s001.zip › Supplementary Figure S3.pptx]

## Slide 1
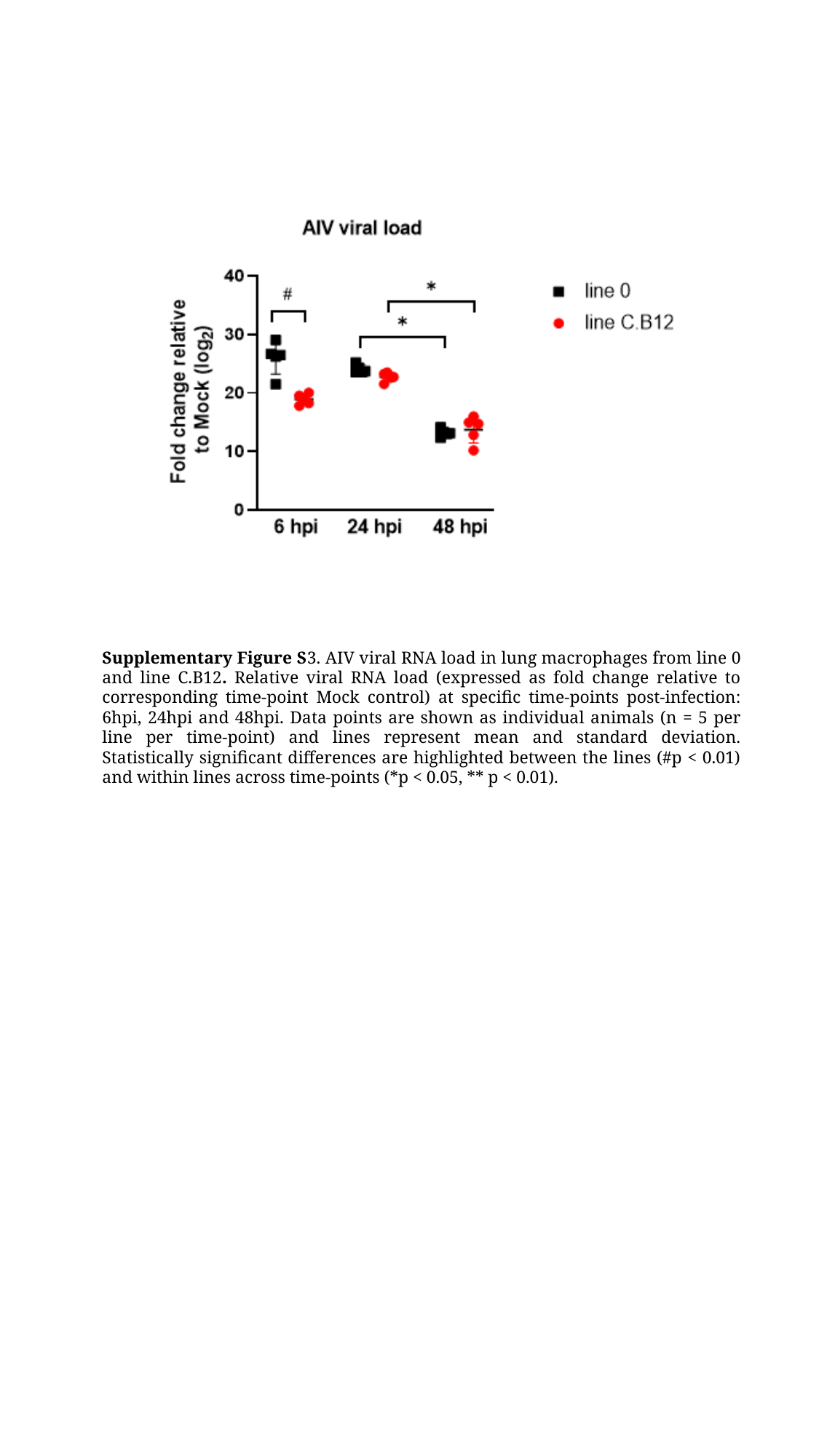

Supplementary Figure S3. AIV viral RNA load in lung macrophages from line 0 and line C.B12. Relative viral RNA load (expressed as fold change relative to corresponding time-point Mock control) at specific time-points post-infection: 6hpi, 24hpi and 48hpi. Data points are shown as individual animals (n = 5 per line per time-point) and lines represent mean and standard deviation. Statistically significant differences are highlighted between the lines (#p < 0.01) and within lines across time-points (*p < 0.05, ** p < 0.01).
