# Supplementary material for: Comparative Analysis of Different Inbred Chicken Lines Highlights How a Hereditary Inflammatory State Affects Susceptibility to Avian Influenza Virus
Source: Viruses. 2023 Feb 21;15(3):591. doi: 10.3390/v15030591 (PMC10052641; doi:10.3390/v15030591)
Supplement: Supplementary file 1 [file viruses-15-00591-s001.zip › Supplementary Figure S4.pptx]

## Slide 1
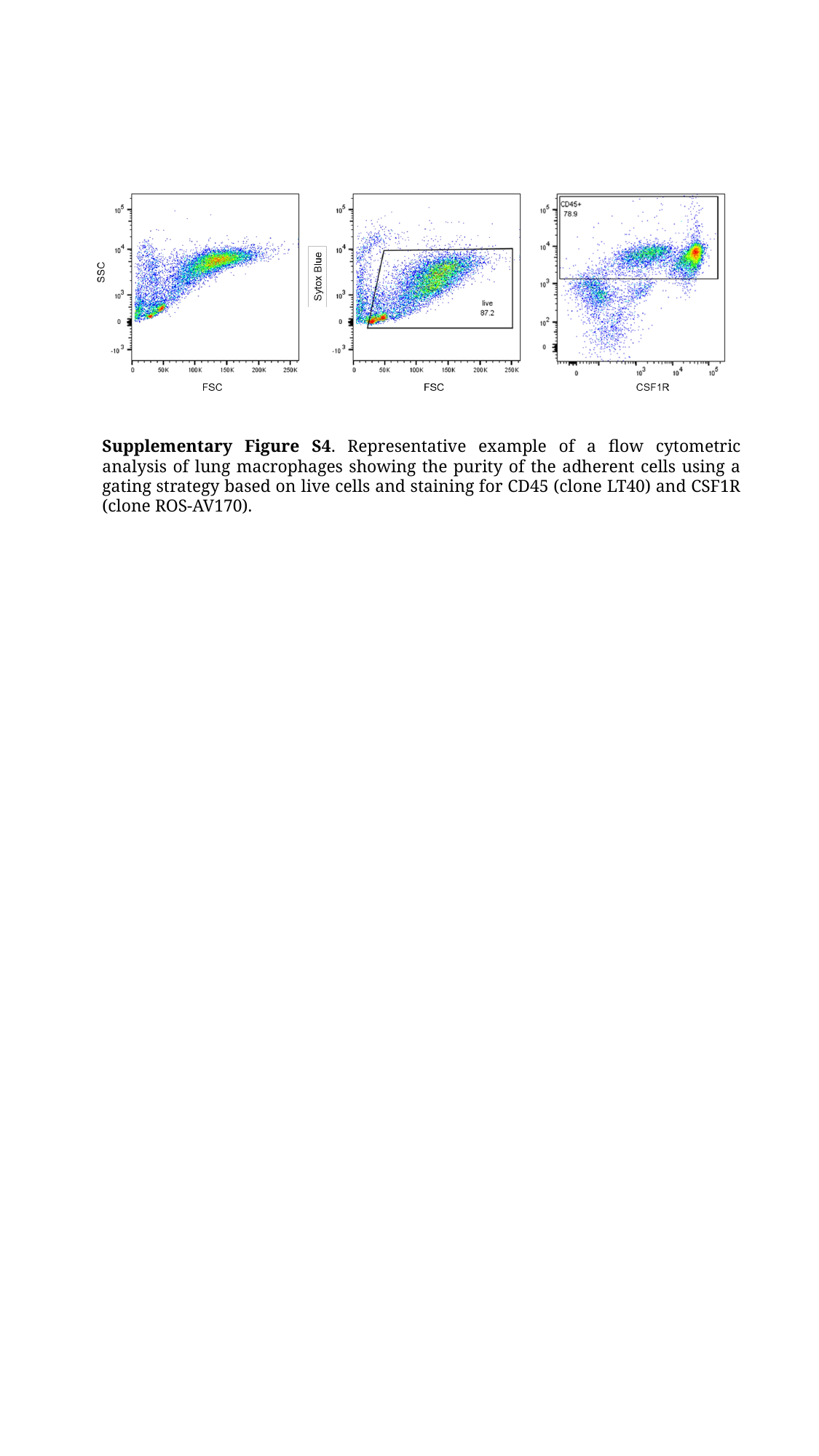

Supplementary Figure S4. Representative example of a flow cytometric analysis of lung macrophages showing the purity of the adherent cells using a gating strategy based on live cells and staining for CD45 (clone LT40) and CSF1R (clone ROS-AV170).
