# Supplementary figures and images for: Comparative Analysis of Different Inbred Chicken Lines Highlights How a Hereditary Inflammatory State Affects Susceptibility to Avian Influenza Virus
Source: Viruses. 2023 Feb 21;15(3):591. doi: 10.3390/v15030591 (PMC10052641; doi:10.3390/v15030591)

## Slide 1
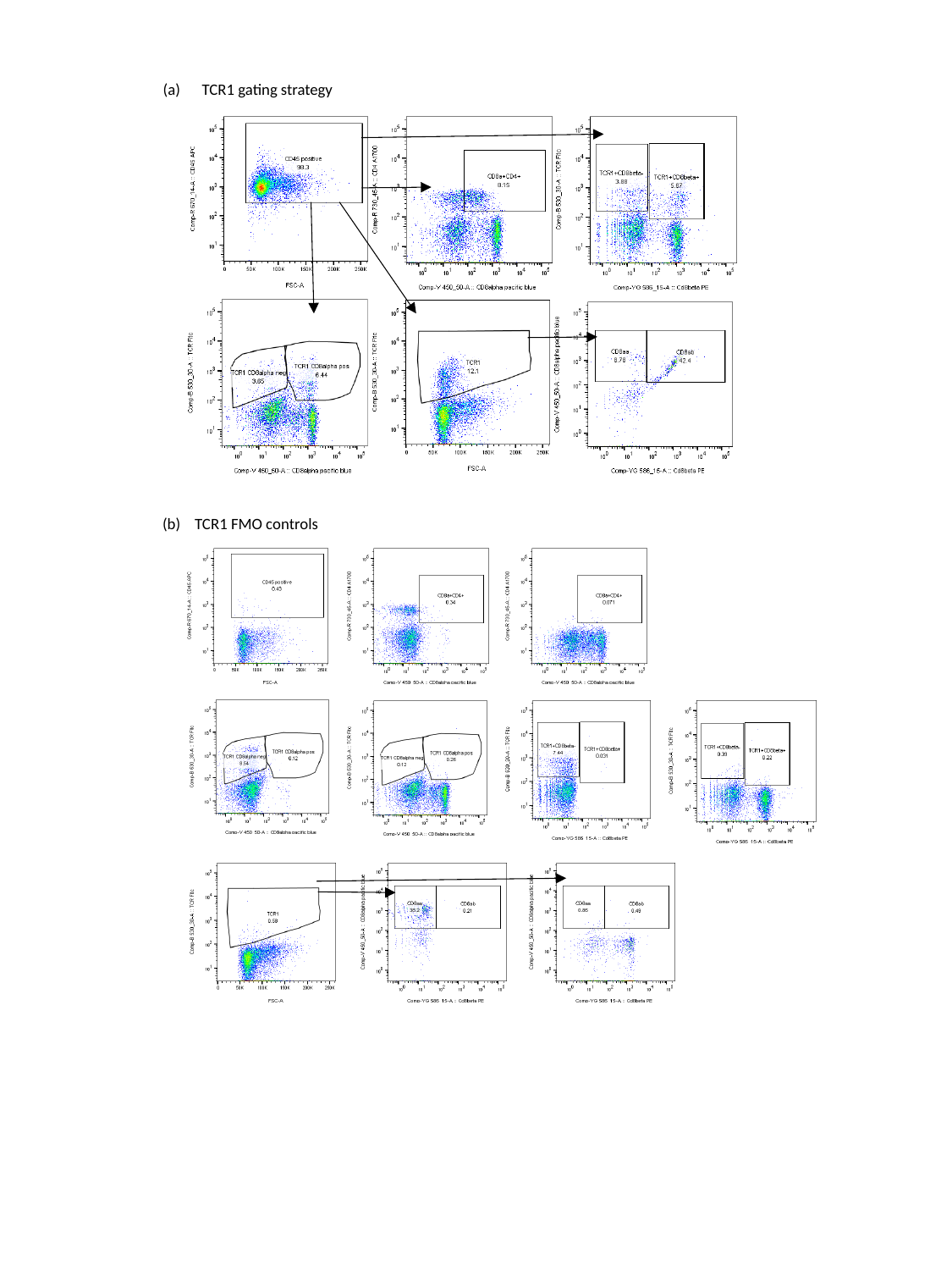

(a) TCR1 gating strategy
(b) TCR1 FMO controls

Supplement: Supplementary file 1 [file viruses-15-00591-s001.zip › Supplementary Figure S2a-b.pptx]
